# Two distinct types of color spreading induced by different luminance and color conditions in static flank transparency displays

**DOI:** 10.3389/fnhum.2023.1087469

**Published:** 2023-02-02

**Authors:** Eiji Kimura

**Affiliations:** Department of Psychology, Graduate School of Humanities, Chiba University, Chiba, Japan

**Keywords:** flank transparency, color spreading, transparency, watercolor illusion, boundary interactions

## Abstract

Flank transparency refers to illusory, phenomenally transparent color spreading induced when narrow colored flanks are added to line segments located within a virtual region. We investigated whether the luminance conditions that induced flank transparency were consistent with predictions based on luminance-contrast-based boundary interactions and the episcotister model for perceptual transparency. Examination of the requirements for the boundary interaction and perceptual transparency revealed that similar luminance conditions are necessary for both; that is, the flank luminance needs to be intermediate between the line and background luminances. We used green and achromatic flanks and systematically varied the luminance of the flanks, line segments, and background. We then asked the observers to rate the subjective certainty of color spreading using a five-point scale. The results showed that the perception of color spreading depended on color as well as luminance conditions. Generally, color spreading was convincing and phenomenally transparent when luminance conditions were consistent with the requirements for boundary interaction and perceptual transparency. In addition, color conditions worked in a facilitatory and inhibitory manner. Moreover, the results revealed that another convincing color spreading could be observed when the flank luminance was lower than the line or background luminance, that is, when the luminance condition for perceptual transparency was not satisfied. The observers’ verbal reports indicated that phenomenal transparency was not evident in this color spreading. Overall, the present findings demonstrate that typical transparent color spreading is not the only one observed in the flank transparency display. Different color and luminance conditions can modulate the phenomenal appearance of color spreading, resulting in distinct types of color spreading.

## 1. Introduction

The apparent color of a region can be significantly affected by the colors of the surrounding or nearby contours. Good examples of the effects can be found in various color-spreading effects such as neon color spreading (Varin, 1971; van Tuijl, 1975; Redies and Spillmann, 1981), watercolor illusion (Pinna et al., 2001), and flank transparency (Wollschläger et al., 2001, 2002).

**FIGURE 1:**
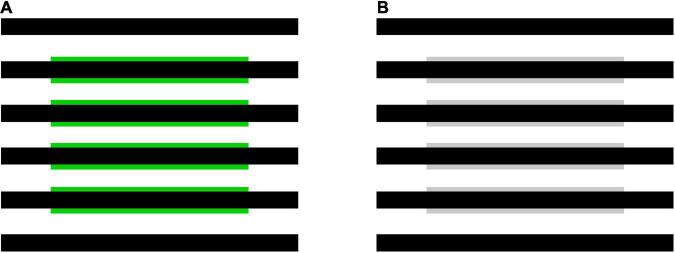
Examples of flank transparency displays (Wollschläger et al., 2001, 2002). **(A)** A stimulus display with green flanks used in Experiment 1, and **(B)** with achromatic flanks used in Experiment 2.

**FIGURE 2:**
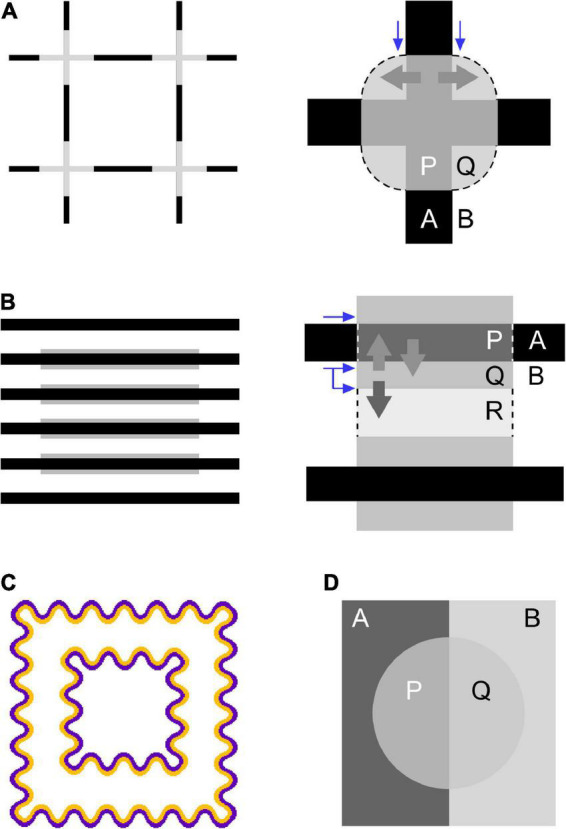
Accounts for color spreading and perceptual transparency in **(A)** neon-color spreading and **(B)** flank transparency displays from the viewpoints of boundary interactions (Grossberg and Mingolla, 1985; Bressan, 1995) as well as the extended episcotister model (Metelli, 1970; Ekroll and Faul, 2002). The left figure demonstrates color spreading illusion in each configuration. The right figure schematically illustrates how color spreading and perceptual transparency are explained. **(C)** An example of a typical orange-purple watercolor display. **(D)** A four-color perceptually transparent display resulting from rotating the episcotister (Metelli, 1970). In the right figures, different letters (A, B, P, Q, and R) designate different regions in the display. Blue and thick gray arrows describe inhibitory effects on boundaries and spreading out of the region’s color, respectively. See the text for details.

Flank transparency refers to illusory color spreading that can be induced by adding narrow colored flanks to line segments located within a virtual shape (the central rectangular region in Figure 1) (Wollschläger et al., 2001, 2002). The illusion produces the percept of a colored transparent filter with illusory contours over the background and line segments within the virtual shape. It has been demonstrated that flank transparency is enhanced in a dynamic display, where the lines are moved relative to the virtual shape (or vice versa) (Wollschläger et al., 2001, 2002). However, the illusion can be demonstrated in a static display (Figure 1). Color spreading in flank transparency appears to be tightly associated with perceptual transparency. This characteristic of flank transparency is shared with neon color spreading but not with the watercolor illusion. Color spreading in the watercolor display is opaque (Pinna et al., 2001; Figure 2C), although color spreading in both flank transparency and watercolor illusion is induced by juxtaposing two differently colored parallel lines.

The present study investigated how luminance conditions among figural components (flanks, line segments, and background) contribute to inducing color spreading and associating color spreading with perceptual transparency in flank transparency displays. First, we describe how color spreading in neon-color spreading and watercolor displays is accounted for from the viewpoint of luminance-contrast-based boundary interactions (Grossberg and Mingolla, 1985; Pinna and Grossberg, 2005). We then extend the account to flank transparency displays. Next, we describe how perceptual transparency in neon-color spreading displays is accounted for using Metelli’s luminance-based episcotister model (Metelli, 1970; Ekroll and Faul, 2002) and extend the account to flank transparency displays. Finally, we argue that luminance conditions suitable for color spreading based on boundary interactions are associated with the requirement for perceptual transparency. Particular luminance conditions can predict perceptually transparent color spreading in flank transparency displays.

### 1.1. Color spreading and boundary interactions

Color spreading in neon-color spreading and watercolor displays has been accounted for by a computational model developed by Grossberg and Mingolla (1985) [see also subsequent studies by Grossberg (1994, 1997), Pinna and Grossberg (2005), and others (e.g., Bressan, 1995)]. The model assumes parallel processing systems, that is, the boundary contour system (BCS) and feature contour system (FCS). The BCS generates invisible (or amodal) boundaries, whereas the FCS fills the space limited by boundaries with color and brightness. Color spreading is assumed to be induced by spatial competition within the BCS. In the model, the strength of a boundary is sensitive to luminance contrast. Moreover, nearby boundaries are assumed to compete with each other. Higher-contrast boundaries inhibit nearby lower-contrast boundaries more than conversely by means of inhibitory interactions between cortical neurons sensitive to similar orientations. For example, in neon-color spreading displays in Figure 2A, the higher-contrast boundary between the black lines (outer limb) and background inhibits the contiguous and lower-contrast boundary between the gray crosses and background (blue arrows). This inhibition makes the lower-contrast boundaries weak and permeable; thus, in the FCS, the color in the region limited by lower-contrast boundaries can flow out into the outer region. In Figure 2A, the gray color of the crosses flows out through permeable boundaries into the background region (thick gray arrows), delimited by illusory contours illustrated by curved arcs (dashed lines). van Tuijl and de Weert (1979) investigated luminance conditions suitable for neon color spreading and reported results consistent with this account based on boundary interactions.

In addition, the BCS can account for the formation of illusory contours in the neon-color spreading display (Figure 2A; Grossberg and Mingolla, 1985). The model assumes that small boundaries are generated at the line ends through a process called end cuts. As shown in Figure 2A, small boundaries are generated at the junctions between the central cross and outer limbs. Because these boundaries have an orthogonal orientation relative to the central cross and outer limb of the figure, they are assumed to be amplified and grouped in a circular shape (dashed lines) *via* boundary interactions in the BCS. Illusory contours play an important role in mediating perceptual transparency in the display, as discussed below.

Pinna and Grossberg (2005) applied the model to watercolor illusion. Color spreading in watercolor displays is also accounted for by inhibitory boundary interactions between the juxtaposed outer and inner contours (OC and IC, respectively). For example, in a typical purple-orange stimulus (Figure 2C), the purple OC has a higher luminance contrast to the background than the orange IC. Pinna and Grossberg (2005) claimed that a higher-contrast OC inhibits lower-contrast IC, even though the OC and IC are parallel to each other. This inhibition weakens the boundary between the IC and background; thus, the orange color of the IC flows out into the region surrounded by the IC. Luminance conditions suitable for color spreading in watercolor displays have been extensively studied (Pinna and Reeves, 2006; Cao et al., 2011; Devinck and Knoblauch, 2012; Coia et al., 2014; Kimura and Kuroki, 2014a), and the results are generally consistent with this prediction, although color contrast has also been indicated to play a critical role in inducing color spreading and determining the spreading color (Devinck et al., 2005; Coia and Crognale, 2014; Kimura and Kuroki, 2014b).

We can further extend this account to flank transparency displays (Figure 2B) and explain color spreading in the display. The higher-contrast boundary between the line segments and background (line-background boundary) inhibits the lower-contrast boundary between the line segments and flanks (line-flank boundary) (straight blue arrow), which accounts for color spreading over the line segments sandwiched between the flanks (thick gray arrows). Additionally, because flanks are usually thin, the higher-contrast line-background boundary can also inhibit the lower-contrast flank-background boundary (bent blue arrow). The two boundaries are not colinear but are located in close proximity and have the same orientation. This inhibition accounts for the color spreading into the background region (thick dark-gray arrow). Moreover, the grouping of small boundaries generated at the line ends of the flanks predicts the generation of illusory contours (dashed lines) that consist of vertical borders of the central rectangular region and delimit color spreading into the background and line segments.

### 1.2. Perceptual transparency and the episcotister model

Ekroll and Faul (2002) investigated luminance conditions for perceptual transparency in neon-color spreading displays using Metelli’s episcotister model (Metelli, 1970). The episcotister model describes the luminance conditions that lead to the impression of transparency in four-color configurations (Figure 2D). A strong impression of transparency can be obtained when the transmittance α, defined in the following equation, is in the range of 0–1,


$$\alpha =\frac{L\,\left(P\right)-L\,\left(Q\right)}{L\,\left(A\right)-L\,\left(B\right)}$$

where L(x) represents the luminance of region x in Figure 2D. Ekroll and Faul (2002) extended Metelli’s episcotister model to neon-color spreading displays (Figure 2A). In the display, an illusory contour, represented by a dashed line, is induced, and region Q is filled with gray color owing to color spreading. Thus, the illusory contour perceptually separates region Q from region B and generates an implicit X junction (Watanabe and Cavanagh, 1993). The model can be applied to the display if we treat region Q as a different region from background B, even if L(Q) = L(B). Ekroll and Faul (2002) demonstrated that the extended model can predict the goodness of perceptual transparency in a dynamic version of neon-color spreading displays. Because L(Q) = L(B) in the neon-color spreading display and 0 < α < 1, the luminance condition for perceptual transparency can be summarized as L(A) < L(P) < L(B) or L(A) > L(P) > L(B). In other words, for color spreading to be perceptually transparent, the central cross must have intermediate luminance relative to the outer limb and background. Ekroll and Faul (2002) further extended the model to three-dimensional cone excitation codes to account for perceptual transparency in chromatic neon-color spreading displays.

We further extend the episcotister model to flank transparency displays (Figure 2B). The model predicts that when the following condition is satisfied, region P ∪ Q appears transparent (Figure 2B),


$$0<\alpha \,\left(\mathrm{line}\right)=\frac{L\,\left(P\right)-L\,\left(Q\right)}{L\,\left(A\right)-L\,\left(B\right)}<1$$

where α(line) represents the transmittance of region P ∪ Q, which includes line segments and flanks. Because L(P) = L(A) in this flank transparency display, the luminance condition for perceptual transparency can be summarized as L(A) < L(Q) < L(B) or L(A) > L(Q) > L(B). In other words, the luminance of the flanks, L(flank), must be intermediate between the luminance of the line segments, L(line), and that of the background, L(bkg), that is, L(line) < L(flank) < L(bkg) or L(line) > L(flank) > L(bkg).

Another luminance condition,


$$0<\alpha \,\left(\text{bkg}\right)=\frac{L\,\left(Q\right)-L\,\left(R\right)}{L\,\left(A\right)-L\,\left(B\right)}<1$$

may be considered for perceptual transparency of region Q ∪ R, where α(bkg) represents the transmittance of region Q ∪ R, which includes flanks and background. This can be the condition for color to spread into the background, although the geometric relationship between the transparent layer and underlying surface is somewhat peculiar. For the above luminance condition to hold for region Q ∪ R, we need to assume that line segments A and P and flank Q belong to the same object, forming a shape like a silhouette of a rolling pin. Nonetheless, as L(R) = L(B) in the display, an interesting relationship holds between α(line) and α(bkg); that is, α(bkg) = 1–α(line). Thus, whenever 0 < α(line) < 1, 0 < α(bkg) < 1 also holds true. Therefore, the episcotister model could predict color spreading with transparency over line segments and the background region (i.e., P ∪ Q ∪ R).

### 1.3. Association between boundary interaction and perceptual transparency

Notably, luminance conditions suitable for perceptual transparency are associated with the requirement for boundary interactions. As described above, the extended episcotister model predicts good transparency in flank transparency displays when L(line) < L(flank) < L(bkg) or L(line) > L(flank) > L(bkg). Whenever these luminance relations hold, the line-background boundary has the highest contrast among the three boundaries, thereby enabling the line-background boundary to render both line-flank and flank-background boundaries weak and permeable. This analysis predicts that color spreading over the background and line segments would be highly correlated; that is, if one is observed, the other would also be observed.

In summary, transparent color spreading in flank transparency displays can be explained in terms of luminance conditions. The analysis based on boundary interactions (Grossberg and Mingolla, 1985; Bressan, 1995) predicts that when the relation L(line) < L(flank) < L(bkg) or L(line) > L(flank) > L(bkg) holds, color spreading from the flanks to the line segments and background would be observed. At the same time, an analysis based on the extended episcotister model (Metelli, 1970; Ekroll and Faul, 2002) predicts that color spreading would appear transparent. We tested these predictions in this study. Specifically, α(line) can be a compact index for the luminance conditions suitable for color spreading and perceptual transparency. Thus, we investigated whether convincing color spreading would be found in the range of 0 < α(line) < 1 in flank transparency displays. Moreover, we investigated whether color spreading would be observed only within these luminance conditions by systematically manipulating the luminance of the flanks, line segments, and background.

To quantify the color spreading in flank transparency displays, we used subjective confidence ratings rather than more objective tasks such as color matching. At first, we attempted to use matching tasks as they have been used in previous studies on flank transparency and watercolor effects (e.g., Wollschläger et al., 2002; Devinck et al., 2005; Reeves et al., 2013). However, in our stimuli, color matching was sometimes very difficult, particularly when transparent color spreading was observed over the black background or black line segments. In those situations, the color of the illusory spreading differed in phenomenal appearance from that of the matching stimulus. The spreading color generally appeared as a transparent veil, whereas the matching color appeared more opaque and solid. Because of this difference, an acceptable match was not always possible by manipulating the luminance and chromaticity of the matching stimulus. Based on these preliminary observations, we adopted the rating procedure. Reeves et al. (2013) reported that when both subjective rating and objective matching were possible, substantial agreements could be observed between the two for the watercolor effect.

## 2. Experiment 1

Experiment 1 investigated the aforementioned prediction using color flanks (green) in the flank transparency display.

### 2.1. Materials and methods

#### 2.1.1. Observers

Six observers participated in Experiment 1. All observers had normal or corrected-to-normal visual acuity and normal color vision as assessed using Ishihara pseudoisochromatic plates. They were naïve to the purpose of the experiments. Prior to the experiment, the observers provided informed consent after a thorough explanation of all the procedures. The experiments were conducted in accordance with the Declaration of Helsinki and approved by the Human Research Ethics Committee of Chiba University.

#### 2.1.2. Apparatus and stimuli

The stimuli were displayed on a 22-inch color CRT monitor (TOTOKU CV921X; TOTOKU Electric Corporation, Tokyo, Japan) with a spatial resolution of 1280 × 1024 pixels and a refresh rate of 100 Hz. The intensity of each phosphor was manipulated with an 8-bit resolution. Spectroradiometric calibration was performed on three phosphors of the monitor using a Minolta CS-1000 spectroradiometer and an LS-100 luminance meter. The observer’s head was stabilized using a chin and forehead rest to maintain a viewing distance of 57.3 cm. The experiments were conducted in a dark room.

**FIGURE 3:**
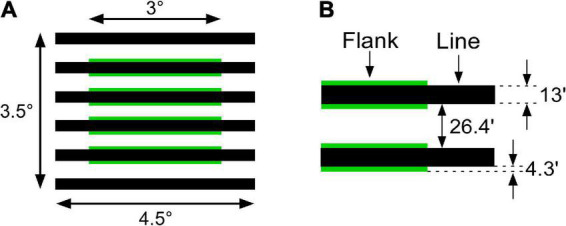
Spatial dimensions of flank transparency displays. **(A)** Overall configuration of the stimulus. **(B)** Spatial details of the line segments and flanks.

The flank transparency display was composed of six horizontal line segments (4.5° in length and 13′ in width). Narrow green flanks (3° in length and 4.3′ in width) were juxtaposed with the four central line segments in a virtual rectangular region (Figure 3). Green flanks were used to obtain a larger luminance range. Line segments were thicker (13′ in width) than those used in previous studies (Wollschläger et al., 2001, 2002) to investigate the color spreading over line segments. Line segments were placed 26.4′ apart from each other. The chromaticity coordinates of the achromatic stimuli were CIE *x* = 0.296 and *y* = 0.324, and those of the green stimuli were CIE x = 0.285 and y = 0.595. The background subtended 5.6° × 5.6°.

Stimulus luminance was manipulated in four luminance conditions: white-background, black-background, white-line, and black-line conditions (Table 1). In each condition, the luminance of the line segments or background was fixed at the maximum (white) or minimum (black) value, and the luminance of the other stimulus components was manipulated. The luminance of the background or line segments was varied in four steps and that of the flanks was varied in eight or nine steps. Thus, each of the four luminance conditions included 32 or 36 luminance combinations. The luminances of different stimulus components were chosen to maximize the monitor gamut. After we measured the black-line and white-background conditions, we changed the settings of the CRT monitor. This change reduced the monitor gamut; thus, we changed the luminance values accordingly in the white-line and black-background conditions.

**TABLE 1:** Luminance conditions in Experiment 1.

Conditions	L(bkg)	L(line)	L(flank)
Black-line	5.0–91.6 (4 steps)	0.0	0.59, 8.64, 16.6, 24.8, 32.8, 40.7, 48.4, 56.6, 64.6 (9 steps)
White-line	0.0–63.9 (4 steps)	81.2	0.71, 8.71, 16.2, 24.2, 31.8, 39.8, 47.5, 56.1 (8 steps)
Black-background	0.0	5.2–81.2 (4 steps)	0.71, 8.71, 16.2, 24.2, 31.8, 39.8, 47.5, 56.1 (8 steps)
White-background	91.6	0.0–64.9 (4 steps)	0.59, 8.64, 16.6, 24.8, 32.8, 40.7, 48.4, 56.6, 64.6 (9 steps)

L(bkg), L(line), and L(flank) represent the luminance of the background, line segments, and flanks, respectively.

**FIGURE 4:**
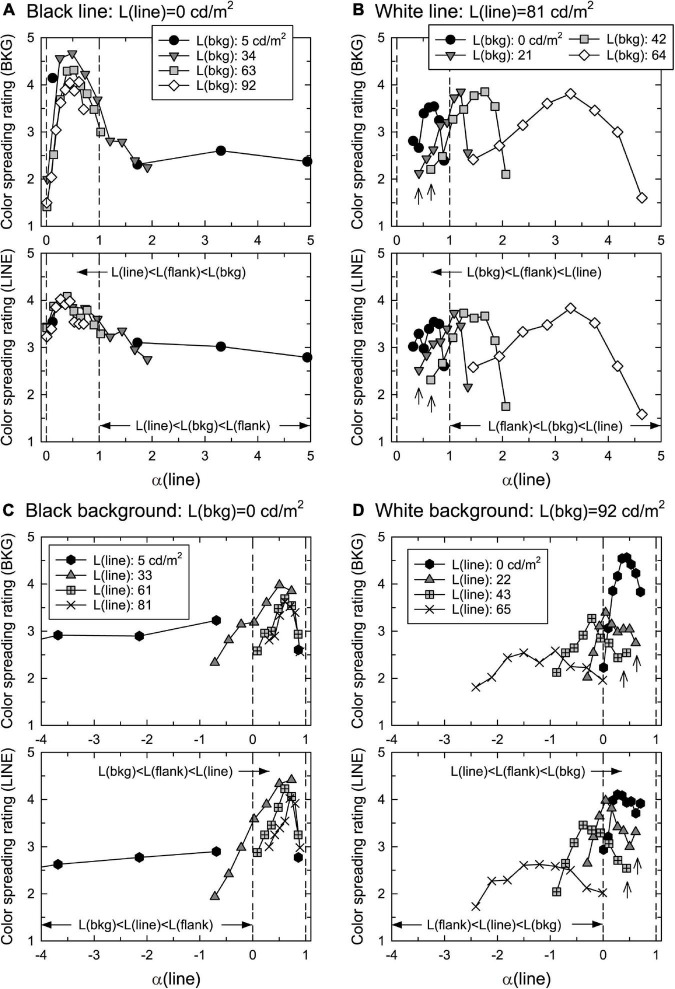
Mean confidence ratings of color spreading as a function of α(line) in **(A)** black-line, **(B)** white-line, **(C)** black-background, and **(D)** white-background conditions. Top and bottom figures in each luminance condition show the rating of color spreading over the background and line segments, respectively. Two vertical dashed lines in each panel show the range of 0 < α(line) < 1. The upward arrows in panels **(B,D)** indicated the ratings that were not high even when α(line) was around 0.5.

To test the replicability of the results, several luminance combinations were duplicated. That is, L(bkg) = 0 in the white-line condition (solid circles shown in Figure 4B) was the same as L(line) = 81.2 in the black-background condition (Xs in Figure 4C), and L(bkg) = 91.6 in the back-line condition (open diamonds in Figure 4A) was the same as L(line) = 0 in the white-background condition (solid hexagons in Figure 4D). These were the same luminance combinations but were measured in different experimental sessions.

#### 2.1.3. Procedure

The observers performed two rating tasks: one regarding color spreading over the background and the other regarding color spreading over the line segments. They were instructed to rate the strength of their subjective confidence that the color of the flanks appeared to spread over the designated regions using a monopolar five-point scale (1–5, with a higher rating indicating higher confidence). It was emphasized that they were not to rate the colorfulness or saturation of the spread color. They were also instructed to use a rating of 0 when some stimulus components (e.g., flanks) were undistinguishable from others (e.g., background); thus, they could not rate the strength of the color spreading. In each trial, a fixation cross (0.5° × 0.5°) was presented at the center of the monitor. The observer’s key press presented the stimulus at the center of the monitor, replacing the fixation cross. There was no time limit to the rating task.

Each luminance condition was blocked during the measurement. In the black-background and white-background conditions, observers were dark-adapted for at least 5 min at the beginning of each daily session. In the white-background condition, the observers were light-adapted to the background for 2 min. In each condition, all luminance combinations (combinations of four line luminances and eight or nine flank luminances) were presented four times in pseudorandom order in each experimental block. Rating tasks for the background and line segments were conducted in different blocks. The order of the blocks was counterbalanced across the observers.

The background luminance varied in the black-line and white-line conditions. Thus, the measurement for each background luminance was blocked, and all background luminances were tested in each daily session in ascending order to maintain the adaptation level. Observers were dark-adapted for at least 5 min at the beginning of each daily session. When the background luminance was changed, the observers were light-adapted to the new background for 2 min before the measurement. In each block, the luminance of the line segments and background was fixed, and all eight or nine flank luminances were presented four times in pseudorandom order. In these luminance conditions, rating tasks for the background and line segments were conducted in consecutive blocks. The order of the rating tasks was counterbalanced across the observers.

For all four luminance conditions, measurements were repeated twice on different days. Thus, all luminance combinations in each condition were rated eight times for each observer. Before the experiment, typical flank transparency displays (see Figure 1A), in which α(line) had intermediate values in the range of 0–1, were shown to observers. All observers confirmed that they saw transparent color spreading in the displays. Before each experimental block, observers had as many practice trials as they wanted to familiarize themselves with the stimulus and task. In the practice trials, all stimuli that would be used in the following experimental block were presented. The observers were instructed to use all rating values for those stimuli.

### 2.2. Results and discussion

Figure 4 shows the mean confidence rating (averaged across different observers) of color spreading over the background and line segments in the four luminance conditions. To closely examine the results in the range of 0 < α(line) < 1, the range of the horizontal axis was limited to the nearby range such that some data points were out of the range in the black-line and black-background conditions. Supplementary Figures 1, 2 show all mean ratings with the 95% confidence interval as a function of flank luminance. The stimuli with α(line) in the range of 0–1 are called “in-the-range” stimuli, and others “out-of-range” stimuli.

The results for the black-line and black-background conditions show that the ratings were highly dependent on α(line) (Figures 4A, C). Regardless of the background luminance, L(bkg), in the black-line condition, or the line luminance, L(line), in the black-background condition, the ratings for the background and line segments were high for in-the-range stimuli and peaked at similar intermediate values. The 95% confidence intervals for stimuli around the peak are generally located above the middle value of the rating scale (3) (Supplementary Figures 1A, B, 2A, B). These results indicate that convincing color spreading was reliably observed with those α(line) values. The ratings decrease when α(line) is close to zero [that is, L(line) = L(flank)] or one [L(bkg) = L(flank)]. Moreover, the ratings for the background and line segments were highly correlated [*r* = 0.718 [*t*(34) = 6.04, *p* < 0.001] in the black-line condition and *r* = 0.967 [*t*(30) = 20.78, *p* < 0.001] in the black-background condition], although the ratings for the line segments were slightly higher around α(line) = 0, particularly in the back-line condition. As described in the procedure, phenomenal observations by the observers confirmed that color spreading is perceptually transparent for in-the-range stimuli (Figure 1A shows an example of such stimuli). Overall, the results are consistent with the predictions based on the analysis of the boundary interaction and generalized episcotister model.

However, the results were different for the white-line and white-background conditions. First, convincing color spreading was reported for out-of-range stimuli, that is, even when the luminance condition was not appropriate for transparency, particularly in the white-line condition. Second, the ratings were sometimes not high for in-the-range stimuli even when α(line) was around 0.5 (the data indicated by the upward arrows in Figures 4B, D) (see also Supplementary Figures 1C, D, 2C, D). It should be noted that the results for the replicated luminance combinations were similar to those in the black-line and black-background conditions. Namely, solid circles in the white-line condition (Figure 4B) and Xs in the black-background condition (Figure 4C) represent the results for the same luminance combinations and the same for solid hexagons in the white-background condition (Figure 4D) and open diamonds in the black-line condition (Figure 4A). They showed similar dependencies on α(line), indicating that changes in the criterion of color-spreading rating are an unlikely reason for these different results. As in the previous conditions, the ratings for the background and line segments were highly correlated [*r* = 0.942 [*t*(30) = 15.37, *p* < 0.001] in the white-line condition and *r* = 0.889 [*t*(34) = 11.34, *p* < 0.001] in the white-background condition].

The finding that the ratings of color spreading were low even when α(line) was around 0.5 might be accounted for by considering the color condition. As the episcotister model had been extended to three-dimensional cone excitation codes (Ekroll and Faul, 2002), we derived α(line) for the L, M, and S cones [α_L_(line), α_M_(line), α_S_(line), respectively]. The results showed that α_L_(line) and α_M_(line) were similar in size to α(line) defined in terms of luminance. When α(line) was in the range between 0 and 1, α_L_(line) and α_M_(line) were in or close to the range. However, α_S_(line) was not because the flank was green. α_S_(line) was greater than 1 when L(bkg) was greater than 0 cd/m^2^ in the white-line condition and less than zero when L(line) was greater than 0 cd/m^2^ in the white-background condition. This violation of the condition for perceptual transparency in α_S_(line) could have resulted in poor transparency and color spreading. If this reasoning is correct, making the stimulus display achromatic would lead to convincing color spreading with transparency even under the same luminance conditions. This prediction was tested in Experiment 2.

We will discuss the convincing color spreading for out-of-range stimuli after examining the results of Experiment 2.

## 3. Experiment 2

Experiment 2 investigated color spreading in the flank transparency display when all stimulus components in the display, that is, the flanks, line segments, and background, were rendered achromatic (Figure 1B). All stimulus components had the same chromaticity coordinates; thus, α(line) values calculated for the three types of cones were also the same.

### 3.1. Materials and methods

The same methods as those in Experiment 1 were used, except for the following: Five of the six observers in Experiment 1 participated in Experiment 2 (one observer was unavailable). The flank was made achromatic (CIE *x* = 0.296, *y* = 0.324), and only the white-line and white-background conditions were tested, where some of the color-spreading ratings were low for in-the-range stimuli and high for out-of-range stimuli. The same luminance steps were used for the white-line and white-background conditions (Table 2). The rating task was conducted only for color spreading over the background because the ratings for the background and line segments were highly correlated in Experiment 1.

**TABLE 2:** Luminance conditions in Experiment 2.

Conditions	L(bkg)	L(line)	L(flank)
White-line	0.0–63.9 (4 steps)	81.2	0.71, 8.71, 16.2, 24.2, 31.8, 39.8, 47.5, 56.1 (8 steps)
White-background	81.2	0.0–63.9 (4 steps)	0.71, 8.71, 16.2, 24.2, 31.8, 39.8, 47.5, 56.1 (8 steps)

### 3.2. Results and discussion

**FIGURE 5:**
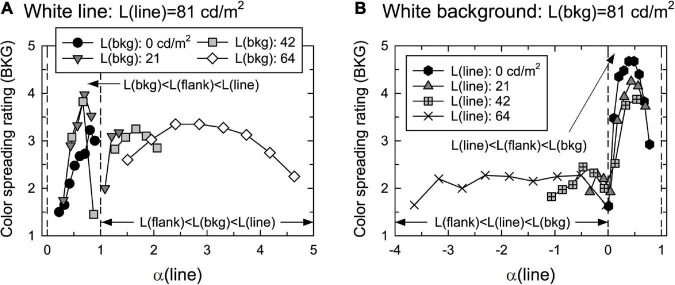
Mean confidence ratings of color spreading over the background as a function of α(line) in **(A)** white-line and **(B)** white-background conditions. Other aspects are the same as those in Figure 4.

Figure 5 shows the mean confidence rating (averaged across different observers) of color spreading over the background in the white-line and white-background conditions. Supplementary Figure 3 shows all the mean ratings with the 95% confidence interval as a function of flank luminance.

With achromatic flanks, the rating of color spreading was dependent on α(line): convincing color spreading was found for in-the-range stimuli, particularly when α(line) had intermediate values in the range of 0–1 in both the white-line and white-background conditions. The 95% confidence intervals for stimuli around the peak are located above the middle value of the rating scale (3) in the white-background condition (Supplementary Figure 3B), although the intervals are wider in the white-line condition (Supplementary Figure 3A). As in Experiment 1, all observers confirmed that color spreading was perceptually transparent for typical in-the-range stimuli (Figure 1B shows an example of such stimuli). The rating became impossible when α(line) was close to 1 in the white-line condition (Figure 5A) because L(flank) was close to L(bkg); thus, the flanks were difficult to discriminate from the background in the display. When α(line) was zero or negative in the white-background condition (Figure 5B), the ratings were low, and the color spreading was not convincing.

Convincing color spreading in in-the-range stimuli suggests that the invalid color conditions for perceptual transparency can suppress color spreading as well as perceptual transparency in flank transparency displays. Moreover, a comparison between the results with achromatic flanks (Figures 5A, B) and those with green flanks (Figures 4B, D) indicates that color conditions are also important for facilitating color spreading in flank transparency displays. With green flanks, relatively high ratings were obtained when α(line) was around 1 [that is, L(flank) = L(bkg)] in the white-line condition and α(line) was around 0 [that is, L(flank) = L(line)] in the white-background condition (Figures 4B, D). These results suggest that color contrast is necessary for spreading color in these stimuli.

**FIGURE 6:**
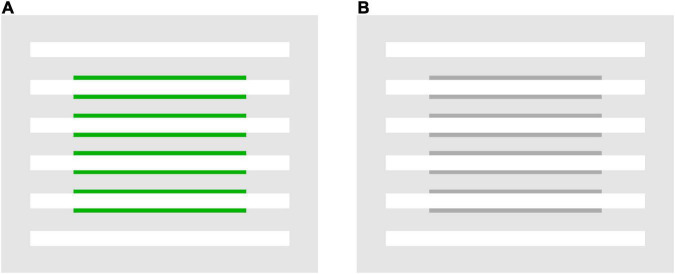
Examples of flank transparency stimuli in which α(line) is outside the range of 0–1 but still convincing color spreading is observed. **(A)** A stimulus display with green flanks and **(B)** with achromatic flanks.

Furthermore, the results showed that, with achromatic and green flanks, convincing color spreading was reported for out-of-range stimuli in the white-line condition. Moreover, color spreading in out-of-range stimuli appears different from that in in-the-range stimuli. All observers confirmed that perceptual transparency was not evident for typical out-of-range stimuli shown after the rating experiments. We cannot provide the results of systematic testing of perceptual transparency because the same observers were not available for further testing. Instead, we demonstrate typical out-of-range stimuli in Figure 6 so that readers can closely inspect their phenomenal appearances. Figure 6A illustrates an out-of-range stimulus with green flanks, and Figure 6B with achromatic flanks. For these stimuli, the color-spreading rating was high. If the reproduction is appropriate, the background and line segments appear greenish in Figure 6A (see also the color-spreading ratings in Figure 4B). However, the impression of transparency is very weak (compare the apparent transparency of these examples with that of the examples in Figure 1). Moreover, the background appears darker with achromatic flanks in Figure 6B, but the color spreading over the line segments may be much weaker than with the green flanks in Figure 6A. Thus, the impression of transparency is not evident in Figure 6B. With achromatic flanks, color spreading over the line segments may not highly correlate with that over the background.

From the viewpoint of boundary interaction, color spreading in out-of-range stimuli cannot be accounted for by the same inhibitory interaction as in in-the-range stimuli (Figure 2B). In these stimuli, the line-background boundary, which is assumed to inhibit both the line-flank and flank-background boundaries in in-the-range stimuli, had the lowest luminance contrast in most cases [specifically, the lower six of the nine flank luminances in the L(bkg) = 64 cd/m^2^ condition]. Therefore, the inhibition by the line-background boundary cannot be effective. Instead, the line-flank boundary exhibited the highest luminance contrast. This boundary might have inhibited the flank-background boundary of lower contrast, resulting in color spreading over the background region. Color spreading in out-of-range stimuli will be further discussed in the section “4. General discussion.”

## 4. General discussion

The present study investigated how luminance conditions contribute to inducing color spreading and associating color spreading with perceptual transparency in flank transparency displays. The analyses from the viewpoints of luminance-contrast-based boundary interactions (Grossberg and Mingolla, 1985; Pinna and Grossberg, 2005) and luminance-based episcotister model (Metelli, 1970; Ekroll and Faul, 2002) predicted perceptually transparent color spreading over the background and line segments when α(line) was in the range of 0–1 (“in-the-range” stimuli). We tested this prediction. Experiment 1 with green flanks provided mixed results; in some of the luminance conditions (black-line and black-background conditions, Figures 4A, C, respectively), the results were consistent with the prediction, and convincing transparent color spreading was reported for in-the-range stimuli. However, in the other conditions (white-line and white-background conditions, Figures 4B, D, respectively), color-spreading ratings were sometimes low, even when α(line) was intermediate (around 0.5).

Experiment 2, with achromatic flanks, revealed that the inconsistent results were probably due to the violation of the color conditions for perceptual transparency. Examining the episcotister model extended for cone excitation codes showed that the condition for transparency was violated for S cones in Experiment 1. Changing the flank color from green to achromatic, thus satisfying the condition for transparency for all three types of cones, restored the transparent color spreading in in-the-range stimuli. Other results, such as higher color-spreading ratings when the flanks were equiluminant to the background [i.e., α(line) = 1] in the white-line condition (Figure 4B), suggest that color conditions work in a facilitatory as well as inhibitory fashion.

As described in the section “1. Introduction,” satisfying the luminance condition for perceptual transparency [0 < α(line) < 1] in flank transparency displays is equivalent to satisfying the luminance condition for L(line) < L(flank) < L(bkg) or L(line) > L(flank) > L(bkg). From the viewpoint of boundary interactions, this luminance condition indicates that the line-background boundary has the highest contrast. Thus, this boundary can inhibit and render both the line-flank and flank-background boundaries permeable, leading to color spreading over the background and line segments. If we extend this account in such a way that boundary contrast is defined in chromatic and luminance terms, the present findings for in-the-range stimuli can be explained. Overall, boundary interactions can induce color spreading. Simultaneously, perceptual transparency results from both geometrical conditions, such as an implicit X junction (Watanabe and Cavanagh, 1993), and color and luminance conditions satisfying the episcotister constraint.

Moreover, the present study revealed that another type of color spreading could be observed for out-of-range stimuli, particularly in the white-line condition (Figures 4B, 5A). An important characteristic of this color spreading was that perceptual transparency was not obvious (Figure 6), which is consistent with the fact that luminance and color conditions for transparency were not satisfied with these stimuli. The fact that α(line) is greater than 1 indicates that the same boundary interaction as in in-the-range stimuli cannot account for color spreading. In most cases, the line-background boundary had the lowest luminance contrast.

If the luminance-contrast-based boundary interaction also works for these out-of-range stimuli, the highest contrast line-flank boundary strongly inhibits the lowest-contrast flank-background boundary. This inhibition could account for the color spreading in the background region sandwiched between the flanks (Figures 6A, B). However, some results cannot be easily explained using contrast-based boundary interactions. One of the results is the convincing color spreading over the line segments observed in out-of-range stimuli in the white-line condition, particularly with green flanks (Figures 4B, 6A). This color spreading cannot be easily accounted for by the contrast-based boundary interaction because for this color spreading to occur, the line-flank boundary needs to become permeable, although it is the highest contrast boundary in the stimulus, at least in terms of luminance contrast. Another result is poor color spreading when α(line) is more than 1 in the black-line condition (Figure 4A). For these stimuli, although α(line) was sparsely sampled, the contrast conditions were similar to those for out-of-range stimuli in the white-line condition. That is, the line-flank boundary had the highest contrast, and the flank-background boundary had mostly the lowest contrast. The contrast polarity of the out-of-range stimuli in the black-line condition was opposite to that in the white-line condition. However, boundary interaction was assumed to be insensitive to contrast polarity (Grossberg and Mingolla, 1985; Pinna and Grossberg, 2005). Some amendments to this account are required to explain these results. Notably, Coia and Crognale (2014) showed asymmetric effects of luminance increments and decrements on color spreading in watercolor displays. These effects were also dependent on color conditions. Moreover, the phenomenal observation in Figure 6 that color spreading over the line segments was much weaker with achromatic flanks (Figure 6B) than with green flanks (Figure 6A) suggested that color contrast plays a critical role in this non-transparent color spreading.

**FIGURE 7:**
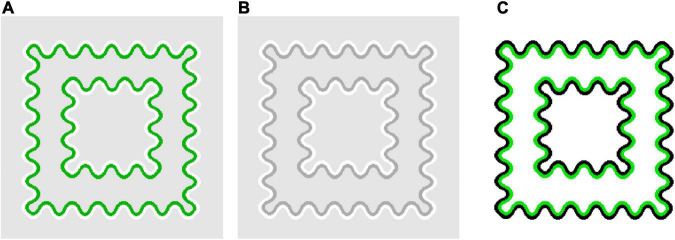
Examples of watercolor stimuli in which the particular luminance and color conditions found in flank transparency displays are applied. In **(A,B)**, the IC and OC colors were set to the colors for which non-transparent color spreading was observed in the flank transparency display (Figures 6A,B, respectively). In **(C)**, the IC and OC colors were set to the colors for which typical perceptually transparent was observed in the flank transparency display (Figure 1A).

We are unable to provide a conclusive explanation of how this non-transparent color spreading is induced. However, it may be worthwhile to show that the luminance and color conditions found in flank transparency displays are generalizable to watercolor displays. Specifically, the luminance and color conditions that induce transparent and non-transparent color spreading in flank transparency displays also produce phenomenally different color spreading in watercolor displays (Figure 7). In Figures 7A, B, the IC and OC colors of the watercolor display are set to the colors of the flanks and line segments of the out-of-range stimuli in Figures 6A, B, respectively. In these figures, readers would observe bidirectional assimilative color spreading [Takashima (2008) reported a similar bidirectional spreading using achromatic stimuli as the “sumi painting” effect]. The corridor region surrounded by the IC appears darker (and slightly greenish in Figure 7A), whereas the central square region surrounded by the OC appears lighter. The bidirectional color spreading is consistent with the boundary interaction in which the highest contrast IC-OC boundary inhibits both the IC-background and OC-background boundaries of lower contrast. By contrast, in Figure 7C, the IC and OC colors of the watercolor display were set to the colors of the flanks and line segments of the in-the-range stimulus shown in Figure 1A. Here, an ordinary watercolor effect is observed: the corridor region appears greenish (and the central region appears slightly brighter owing to a brightness contrast effect). The color spreading is consistent with the boundary interaction in which the highest contrast OC-background (and/or IC-OC) boundary inhibits the IC-background boundary of lower contrast.

In summary, the present findings, together with additional demonstrations, suggest that phenomenally distinct color spreading can be observed in color-spreading displays depending on the color and luminance conditions. In addition to geometrical conditions, such as boundary orientation and junctions, color and luminance conditions presumably determine which boundary suppresses which and whether proximal color information is decomposed into a transparent layer and a background component. These two processes, that is, boundary interaction and perceptual scission, regulate the phenomenal appearance of color-spreading illusions. Future studies are required to explore suitable color conditions in flank transparency displays and to elucidate further the visual mechanisms underlying distinct types of color spreading.

## Data availability statement

The raw data supporting the conclusions of this article will be made available by the authors, without undue reservation.

## Ethics statement

The studies involving human participants were reviewed and approved by Human Research Ethics Committee of Chiba University. The patients/participants provided their written informed consent to participate in this study.

## Author contributions

The author confirms being the sole contributor of this work and has approved it for publication.
