# Supplementary material for: Two distinct types of color spreading induced by different luminance and color conditions in static flank transparency displays
Source: Front Hum Neurosci. 2023 Feb 2;17:1087469. doi: 10.3389/fnhum.2023.1087469 (PMC9936811; doi:10.3389/fnhum.2023.1087469)
Supplement: Supplementary file 1 [file Presentation_1.pdf]

Supplementary Material

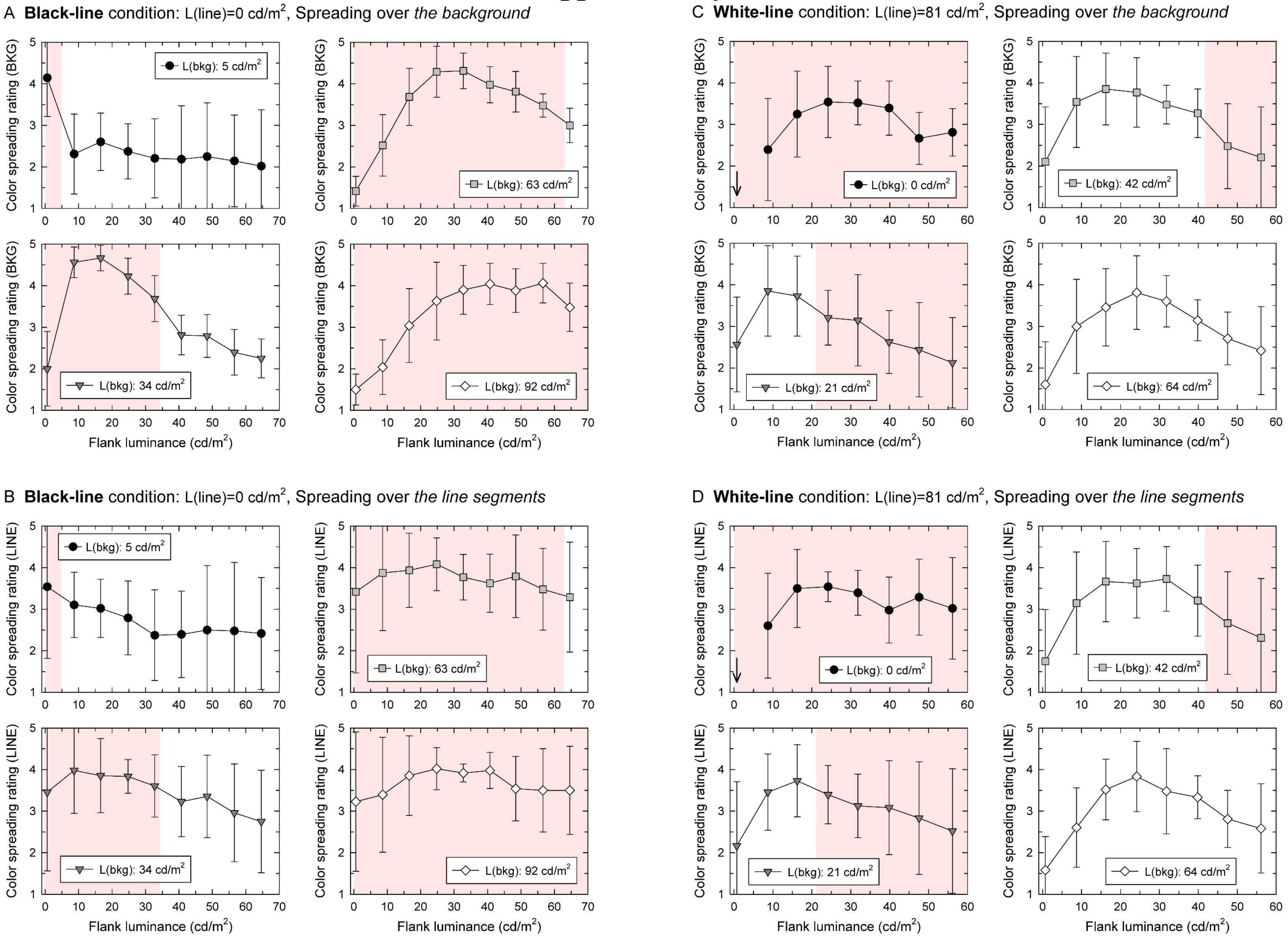

Supplementary Figure 1. Mean confidence ratings of color spreading as a function of flank luminance in Experiment 1 (green flanks): The results for color spreading over **(A)** the background and **(B)** line segments in the *black-line* condition, and those over **(C)** the background and **(D)** line segments in the *white-line* condition. Error bars represent the 95% confidence interval. To avoid superimposition of symbols and error bars, the results for different background luminances [L(bkg)] were shown in separate panels. The same symbols are used for different background luminances as those in Fig. 4. The red shaded area in each panel designates the luminance range of $0 < \alpha(\text{line}) < 1$. The downward arrow indicates that the rating is missing for the specific flank luminance. The observers reported that the rating was impossible for that stimulus because the flanks were indiscriminable from the background.

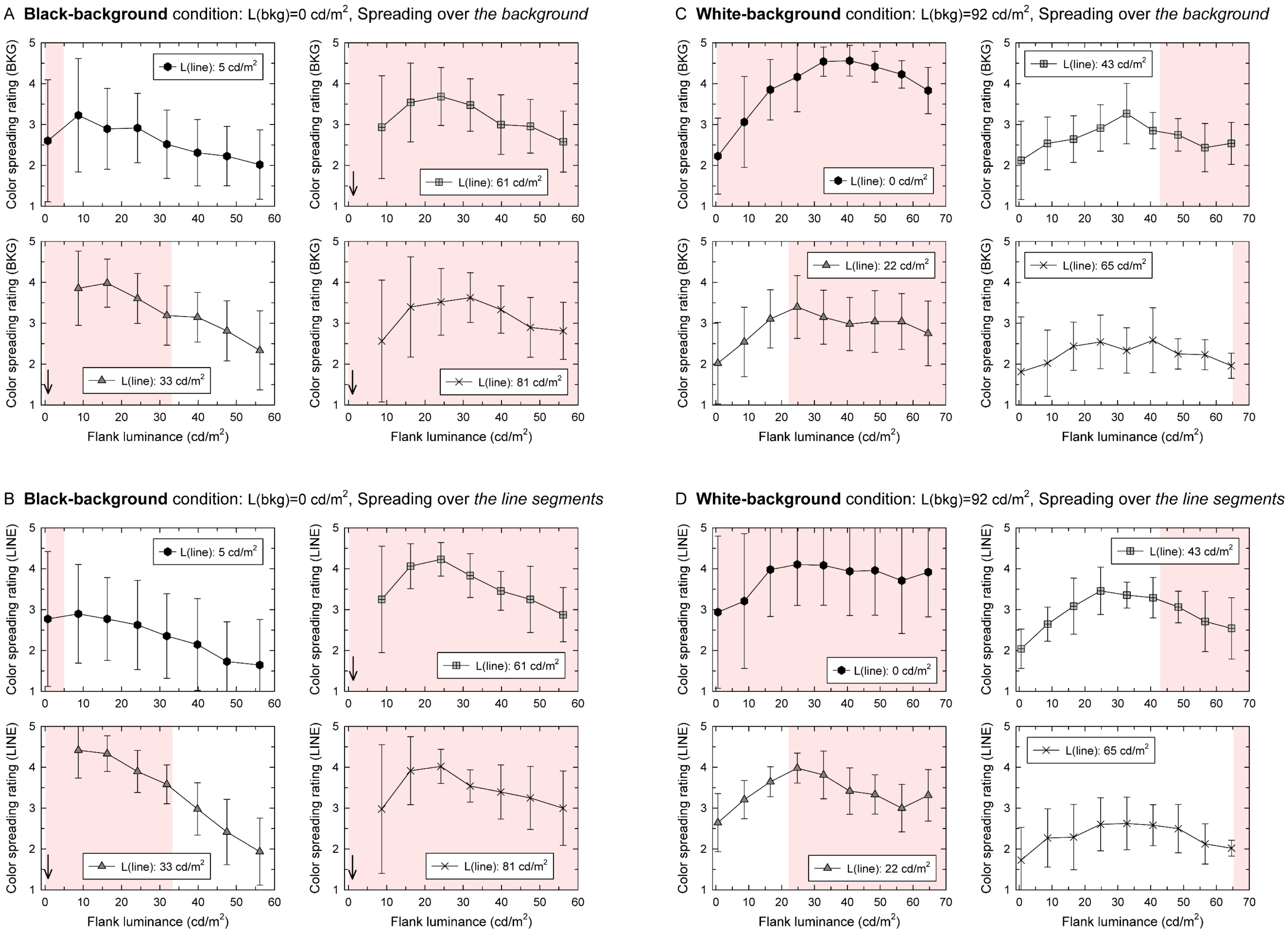

Supplementary Figure 2. Mean confidence ratings of color spreading as a function of flank luminance in Experiment 1 (green flanks): The

results for color spreading over **(A)** the background and **(B)** line segments in the *black-background* condition, and those over **(C)** the background and **(D)** line segments in the *white-background* condition. The results for different line luminances [L(line)] were shown in separate panels for clarity. Other aspects are the same as those in Supplementary Figure 1.

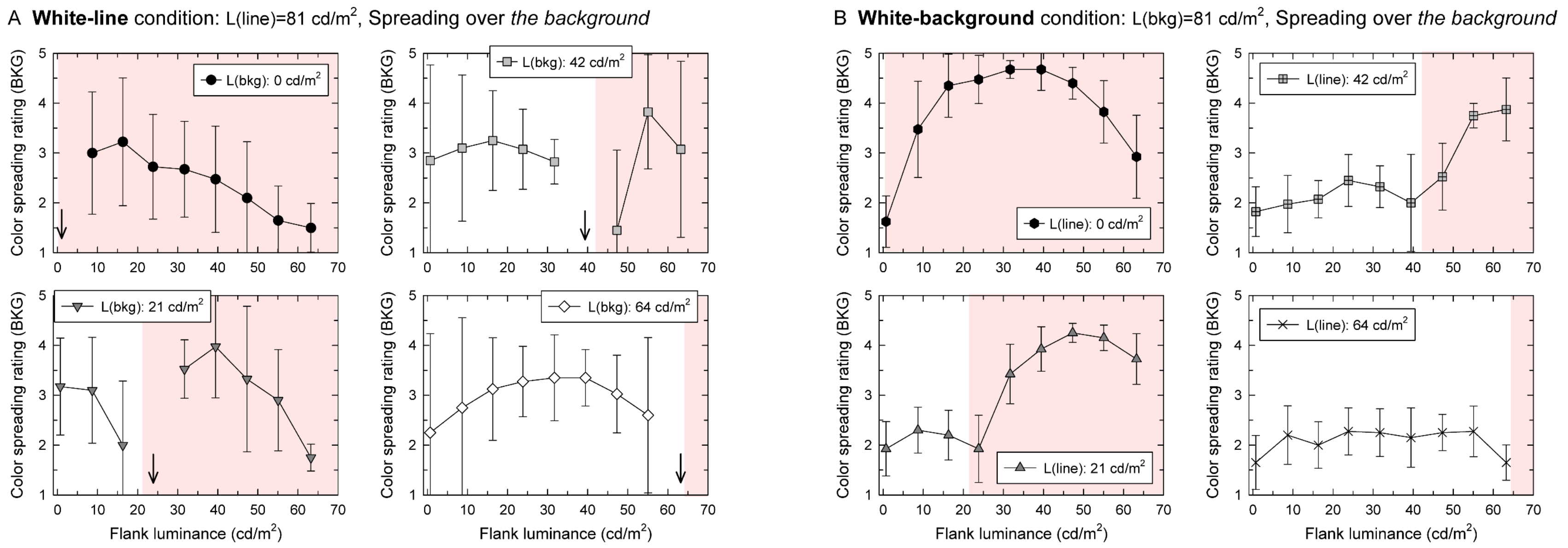

Supplementary Figure 3. Mean confidence ratings of color spreading over the background as a function of flank luminance in Experiment 2 (achromatic flanks): The results for color spreading in **(A)** *white-line* and **(B)** *white-background* condition. The results for **(A)** different background luminances in the white-line condition or **(B)** different line luminances in the white-background condition were shown in separate panels for clarity. Other aspects are the same as those in Supplementary Figure 1.
